# Effects of the National Essential Medicine System in reducing drug prices: an empirical study in four Chinese provinces

**DOI:** 10.1186/2052-3211-7-12

**Published:** 2014-09-17

**Authors:** Yan Song, Ying Bian, Max Petzold, Lingui Li, Aitian Yin

**Affiliations:** State Key Laboratory of Quality Research in Chinese Medicine, Institute of Chinese Medical Sciences, University of Macau, Av. Padre Tomás Pereira Taipa, Macau, 999078 China; Center for Applied Biostatistics, Sahlgrenska Academy, University of Gothenburg, Box 100, S-405 30, Gothenburg, 40530 Sweden; The College of Management, Ningxia Medical University, 1160 Shengli Street, Yinchuan, Ningxia Province 750003 China; Center for Health Management and Policy, Shandong University, 44 Wenhua Xilu, Jinan, Shandong Province 250012 China

**Keywords:** China, National Essential Medicine System, Access to medicines, Drug price, Price index

## Abstract

**Objectives:**

The rapid increase in drug expenditure has become a major source of public criticism in China. In 2009, the National Essential Medicine System (NEMS) was launched in China to control drug prices and improve access to medicines. This study investigated whether and to what extent the prices of essential medicines were reduced after the introduction of NEMS.

**Methods:**

Data were obtained from 149 public primary healthcare centers (PHCs) in four Chinese provinces (Shandong, Zhejiang, Anhui and Ningxia) using a facility-based survey. In total, 10,988 essential medicines were investigated. Individual price differences and a price index were used to measure price changes for three different lists: 2009–2010, 2010–2011, and 2009–2011.

**Results:**

In the comparison between 2009 and 2010, a median decrease of 34.4% [95% confidence interval: 30.4%–39.1%] was observed in drug prices and the number of drug sales increased by 1.5%. The higher the retail price in 2010, the more the drug sales increased compared with 2009 (*χ*^2^ = 75.9, p < 0.01). The drug revenues in 100 of the 149 surveyed PHCs decreased by an average of 39%. Where the available data allowed price changes for 2009–2011 to be assessed, drug prices were reduced significantly in 2010, but a modest decrease was seen in 2011. The Laspeyres index was less than 100 and the Paasche index was larger than the Laspeyres index in 2010 and 2011, which indicated that the frequently prescribed drugs usually had higher prices and any price reduction was milder.

**Conclusions:**

The introduction of NEMS in PHCs in China led to price reductions in essential medicines. However, more-expensive drugs were preferred in the postreform period. Most PHCs had less drug revenue and could encounter financing dilemmas after the implementation of NEMS. Policy options such as improving the compensation mechanism and rational use of drugs should be further promoted in PHCs.

## Introduction

The rapid increase in drug expenditure has become a critical obstacle in accessing health care for the poor and its control is a key objective for healthcare policy makers [1]. China is no exception. There are many reasons for this increased expenditure on drugs. Reasonable factors include more people being treated, more elderly people needing more drugs, increased chronic diseases, and the diffusion of new drugs. In 2010, the number of Chinese aged 60 years and older reached 177 million, which includes 13.3% of the residents of mainland China. This is an increase of 2.9% compared with the same age group in 2000 [2]. The latest National Health Services Survey in China revealed that approximately 20% of the Chinese population had chronic diseases in 2008, which was an annual average of ten million new cases over the last decade [3]. Most potentially irrational factors of increased drug expenditure in China relate to the inflation of drug prices, the increased volume of prescriptions, and the induced demand for physicians to use more expensive drugs [4, 5]. Since the early 1980s, drug revenue has become an essential financial resource for most public healthcare facilities, especially at primary healthcare centers (PHCs) (ranging from 50% to 90% of total revenue) [6]. In contrast to most Western countries, the healthcare provider is the main supplier of pharmaceutical products in China, accounting for 70% of all drugs sold and distributed [7]. The government set prices for basic healthcare below actual cost to keep health care affordable, but allowed a 15% profit margin on drugs to ensure that healthcare facilities survive financially [8]. However, this price-setting approach induced an increase in consumption and overuse of the more expensive or profitable drugs [9].

As such, drug expenditures have been growing by 15% per year and represent almost 50% of total healthcare spending in China, compared with 18% in Organization for Economic Cooperation and Development countries [10]. This disparity is partly because of the 15% drug markup policy (the supplier-induced drug expenditures accounted for 12% to 37% of total medical expenditures) and the severity of irrational use of drugs (nearly 80% of randomly selected prescriptions were for unnecessary antibiotics) [6].

In addition, China’s pharmaceutical distribution industry is extremely fragmented with over 13,000 distributors in 2009. The three largest Chinese distributors accounted for only 22% of the market in 2010 [11]. As a result of this fragmentation, drugs are distributed through several layers of distributors with multiple handoffs before reaching the end-customer, which creates inefficiencies and higher distribution costs in China. Furthermore, most pharmaceutical manufacturers have actively promoted their products using a variety of strategies including hiring numerous salespersons, sending medical representatives to promote prescriptions, or advertising in public media for over-the-counter products. These commercial promotion activities and profits from multiple layers of distribution contribute to a substantial proportion of the total costs of drugs [12].

Chinese households devoted 40%–60% of their out-of-pocket healthcare expenditures to drugs between 1995 and 2008 according to the Chinese Health Statistical Digest [13]. Therefore, the cost of serious illness is a major financial burden for families at the household level. To alleviate Chinese citizens’ burden of expensive medical bills and increase their access to essential medicines, in April 2009 the State Council of China launched the National Essential Medicine System (NEMS) with the goals of cutting the profit link between healthcare facilities, doctors, and drugs, and to improve drug availability, affordability, and rational use [14]. NEMS was initially designed for public primary healthcare facilities, with the intention of extending it to private providers and hospitals.

The NEMS included policies targeting drug selection, production, procurement, distribution, pricing, use and reimbursement. The core of the program was the National Essential Medicine List, which included 205 Western medicines and 102 traditional Chinese medicines (TCMs) [15]. To address local needs, the central government gave authority to provincial governments to supplement the list according to their economic situation and specific needs. Another key policy element of the NEMS was a zero markup policy under which essential medicines were sold to patients at the procurement price, with no profit to healthcare facilities for the sale. Province-based bidding and centralized procurement for essential medicines were developed to simplify the supply chain and strengthen market competition, which were effective in achieving the lowest possible drug prices.

The NEMS has been accepted worldwide as a powerful tool to promote access and rational use of quality drugs. There is substantial evidence that the adoption of the essential medicines concept has contributed to better cost-effectiveness in healthcare systems [16, 17]. This study was to investigate the impact of NEMS on drug prices and to determine whether and to what extent the prices of essential medicines were reduced after the introduction of NEMS.

## Methods

### Study design and sampling

This was a comparative study of pre- and postreform periods in China. The data were collected from 2009 for the prereform period and from 2010 and 2011 for the postreform period. A field survey was conducted to collect the price information of essential medicines. Four Chinese provinces, Shandong, Zhejiang, Anhui, and Ningxia, which represented varying levels of socioeconomic status, were selected as research areas. Shandong and Zhejiang are located in eastern China and represent the developed parts of China. Anhui is part of central China and is an example of a moderately developed region. Ningxia is in northwest China and is an undeveloped region. One hundred and forty-six public PHCs were included through stratified sampling in these four provinces.

The survey in Zhejiang and Anhui was conducted in 2010. It was based on the program “Mid-term Evaluation on Implementation Effects of National Essential Medicine System,” organized by the National Development Reform Committee, China. In each PHC, drugs used in August 2009 and August 2010 with the same strengths and dosage forms were included for price comparison. The surveys in Shandong and Ningxia were conducted in 2011. In each PHC, price comparisons were made for drugs used in June 2009, June 2010, and June 2011 with the same strengths and dosage forms.

### Data collection

A self-compiled questionnaire was used collect price information in the information or purchasing divisions of the PHC. Items included drug name, dosage, form, price, and sales. All the information in 2009, 2010, and 2011 were acquired through the PHCs’ drug-price recording system. The completed forms were checked on a daily basis. A follow-up telephone call was placed if any important information was missing.

### Data analysis

Drugs (same chemical entity) with various strengths and dosage forms were considered as separate drugs in this study. For example, enalapril tablet 10 mg and enalapril tablet 5 mg were considered as two drugs. All compared drugs were the same generic name (and not necessarily from the same producers). The number of drug sales was calculated in accordance with the drug packaging units. For example, norfloxacin’s capsule specification was 0.1 g per tablet, packaging 12 tablets per board, and its sales were calculated in line with boards. In total, 10,988 drugs were analyzed in this research, which included 7769 national essential medicines (NEDs) and 3220 provincial supplemented essential medicines (PEDs). During the analysis, the consumer price index was used to correct 2010 and 2011 prices to 2009 prices to account for deflation. Additionally, all prices were converted to United States dollars (US$) using the mean exchange rate for the year 2009, which was 6.8 Chinese yuan to one US$.

#### Individual price differences

Individual price differences were calculated for each drug before and after NEMS. The comparison was performed as the percentage difference that may appear as a negative or positive value. Compared with the drug price in period 1, the percentage change in drug price in period 2 was calculated using formula (1). Similarly, the percentage change in drug sales in period 2 was calculated using formula (2).
12

Next, the magnitude of the overall price change was calculated for three different lists: 2009–2010, 2010–2011, and 2009–2011. Because individual price differences were abnormally distributed, a median value was used in discussing them. The 95% confidence interval (CI) of the median value was also calculated. A nonparametric test was used to compare price differences between the two periods. A Pearson correlation test was used to test for significant or nonsignificant variations in relationships between prices and sales. The statistical tests were performed with IBM SPSS Statistics software (version 19.0, IBM SPSS, Armonk, NY, USA).

#### Price index

Economic theory defines a price index as reflecting what happens to the overall level of prices in a given period of time. A price with a fixed basket of goods is called a Laspeyres index (*P*^*La*^) in the economics literature, and a price index with a changing basket is called a Paasche index (*P*^*Pa*^) [18]. During a relatively short period (e.g., 10 years), a fixed basket of goods will not change. However, with variation in consumer preferences and social production capacities, the commonly used basket of goods will change. Therefore the application of *P*^*La*^ and *P*^*Pa*^ can indicate the interactive relationship between prices and sales. In this study, all surveyed drugs were regarded as a fixed basket, and each year the drugs ranked in the top 50 according to the number of sales were regarded as a commonly used basket. These two indicators were calculated with formula (3) and (4):
34

where *P*_*1i*_ and *P*_*2i*_ stand for prices of drug *i* in periods 1 and 2, and *Q*_*1i*_ and *Q*_*2i*_ stand for the use of drug *i* in periods 1 and 2. For each price index, a value of less than 100 means that the prices were reduced in a given period of time, and a value of more than 100 means that prices increased in a given period of time.

## Results

### Changes in drug prices

#### Individual price differences

In the comparison between 2009 and 2010, out of 10,988 surveyed drugs, the prices of 9747 (88.7%) drugs were reduced by 39.8% (95% CI, 38.8%–40.0%) and 1057 (9.6%) drugs were increased by 27.5% (95% CI, 25.0%–32.4%). Overall, a median decrease of 34.4% (95% CI, 30.4%–39.1%) was observed in drug prices compared with 2009. The prices of NEDs were down by 35.3% and the prices of PEDs decreased by 32.9%. In each province studied, drug prices showed obvious decreases between 2009 and 2010 with statistical significance (p < 0.01). Details are presented in Table 1. Tables 2 and 3 depict the price changes in essential medicines categorized according to their Clinical Pharmacology Classification from 2009 to 2010. Cardiovascular medicines, which have the second largest market share after antimicrobial medicines in China, decreased by 28.3% in 2010 compared with their prices in 2009. The price decrease was lower than the average level (34.4%) and ranked second to last. Antimicrobial medicines decreased by 46.9% in the corresponding period.

**Table 1:** **Overall price differences for all drugs and for drugs in various subcategories (2009–2010)**

Category		N	Price (in Chinese yuan)			Price chang (%)
			2009	2010	p-value	
Category(1)	Western medicines	7897	4.5	2.1	<0.01	−41.6
	TCMs	3091	10.8	8.1	<0.01	−24.3
Category(2)	NEDs	7768	5.0	2.6	<0.01	−35.3
	PEDs	3220	9.0	5.5	<0.01	−32.9
Region	Shandong	7897	3.5	1.9	<0.01	−40.0
	Zhejiang	3091	7.4	4.1	<0.01	−33.6
	Anhui	7768	5.0	2.2	<0.01	−37.0
	Ningxia	3220	2.6	2.4	<0.01	−4.8
Total		10988	5.7	3.0	<0.01	−34.4

Note: United States dollar = 6.8 Chinese yuan.

p value refers to nonparametric test for statistical difference of prices between the two periods.

TCMs, traditional Chinese medicines; NEDs, national essential drugs; PEDs, provincial essential drugs.

**Table 2:** **Price changes of Western medicines categorized according to Clinical Pharmacology Classification (2009–2010)**

Clinical pharmacology classification	N	Price change (%)
Antiallergics	121	−60.3
Blood-related medicine	317	−52.6
Water-Electrolyte-Acid–base-Balance Medicine	842	−49.2
Antiparasitics	218	−48.9
Urinary system medicine	152	−48.8
Antimicrobial medicine	1643	−46.9
Gynecological medicine	94	−46.7
Vitamin & mineral supplements	519	−43.6
Antipsychotics	70	−42.3
Ophthalmic medicine	177	−40.0
Dermatologic medicine	291	−39.5
Nervous system medicine	298	−39.1
Digestive system medicine	842	−38.9
Respiratory system medicine	333	−37.5
Hormone	489	−34.8
Ear, nose, and throat medicine	38	−29.6
Antipyretic-analgesic and anti-inflammatory medicine	426	−29.4
Cardiovascular system medicine	805	−28.3
Biological medicine	72	72.2
Others	151	−50.0

**Table 3:** **Price changes of traditional Chinese medicines categorized according to Clinical Pharmacology Classification (2009–2010)**

Clinical pharmacology classification	N	Price change (%)
Ophthalmic medicine	62	−35.4
Internal medicine	2309	−25.6
Surgical medicine	199	−25.5
Gynecological medicine	159	−24.2
Orthopedic medicine	288	−11.6
Ear, nose, and throat medicine	74	7.4

#### Trends in drug sales as prices changed

Compared with 2009, the number of drug sales increased by 1.5% (95% CI, 0.0%–12.5%) in 2010 following reductions in drug prices. Price reductions of 40% or more increased the drug sales by about 5.3%. A 20%–40% decrease in price was followed by an increase in drug sales of 4.8%. Price reductions of less than 20% did not have an impact on drug sales. Although the increased sales following the reductions in drug prices were not substantial, they still demonstrated a positive relationship between them (*χ*^2^ = 96.1, p < 0.01). Furthermore, the higher the retail price in 2010, the more the sales increased compared with 2009 (*χ*^2^ = 75.9, p < 0.01) (Table 4). Table 4 shows that the price decreases were relatively low for high-priced drugs. For example, a decrease of 51.8% was observed in the prices of drugs retailing for less than 2yuan (0.3US$), while a fall of 16.3% was observed in the prices of drugs retailing at more than 20yuan (2.9US$). Additionally, drug revenues in 100 of 149 surveyed PHCs reduced by an average of 39% in 2010 compared with the prereform period.

**Table 4:** **Changes in the prices and sales for drugs of different price levels (2009–2010)**

2010 price (in Chinese yuan)	N	Changes in the prices (%)	Changes in the sales (%)	p-value
0-	4189	−51.8	0	<0.01
2-	4420	−32.4	0.1	
10-	1404	−23.6	15.6	
20-	975	−16.3	20.5	

Note: United States dollar = 6.8 Chinese yuan.

p value refers to Pearson correlation test for significant or nonsignificant variations in relationships between price levels and increased sales.

### Further investigation in Shandong and Ningxia in 2011

**Figure 1:**
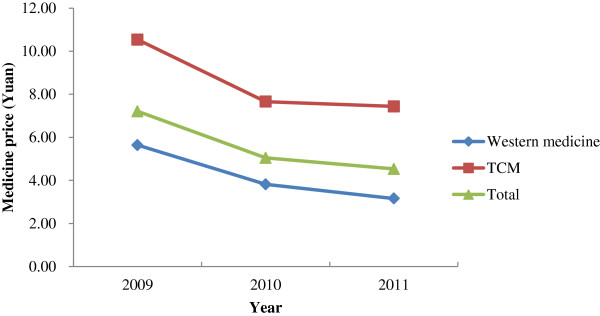
**Evolution of drug prices in Shandong and Ningxia in 2009–2011.**

The price trends of essential medicines in 2011 were investigated further in Shandong and Ningxia. As shown in Figure 1, the results of this additional investigation found that drug prices decreased each year after the implementation of the NEMS (p < 0.01). In most cases, drug prices showed a very large decrease in 2010 and a modest decrease in 2011. In total, a decrease of 40% (95% CI, 34.3%–43.7%) was observed in drug prices between 2009 and 2011. The prices of TCMs and Western medicines showed similar decreasing trends during this period (p < 0.01). The drug revenues in PHCs showed a continuous decline from 2009 to 2011 with statistical significance (p < 0.05).

### Analysis based on price index

**Figure 2:**
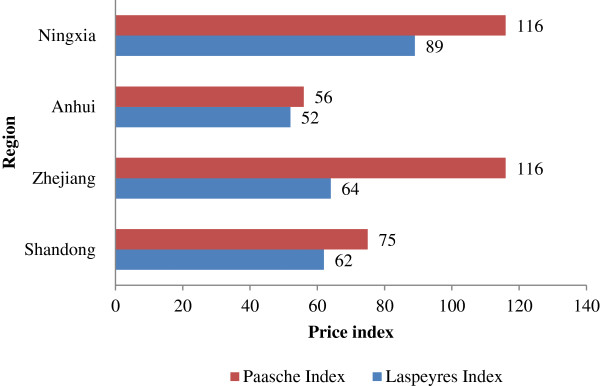
**Laspeyres index and Paasche index in the four provinces studied in 2010.**

**Figure 3:**
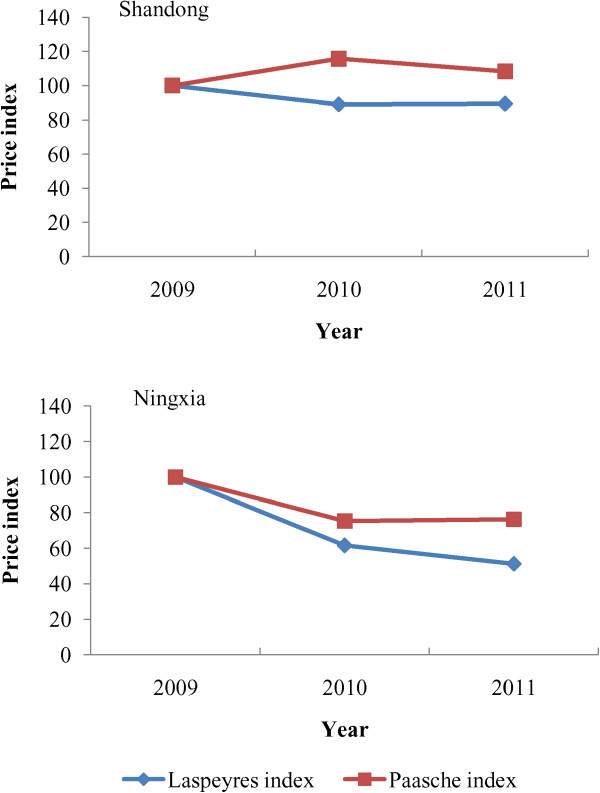
**Laspeyres index and Paasche index in Shandong and Ningxia in 2009–2011.**

Figure 2 shows the values of *P*^*La*^ and *P*^*Pa*^ for essential medicines in the four Chinese provinces studied in 2010. In each province, the value of *P*^*La*^ was less than 100 and showed a decrease in drug prices. The value of *P*^*Pa*^ was always larger than *P*^*La*^ in any condition, which indicated that frequently prescribed drugs usually had higher price levels with milder price reductions. Notably, the values of *P*^*Pa*^ in Zhejiang and Ningxia reached as high as 116 in 2010 and showed a rise of 16% in the frequently prescribed drug prices compared with 2009. The same situation was observed in Shandong and Ningxia in 2011(Figure 3).

## Discussion

### NEMS and changes of drug prices

This study compared the drug prices before and after the implementation of NEMS in four Chinese provinces. The results from the four provinces indicated a median decrease of 34.4% in drug prices in 2010 compared with 2009. Further investigation in Shandong and Ningxia showed that prices continued to drop in 2011. These results are consistent with other research [19, 20]. The Chinese Ministry of Health reported that drug prices in PHCs went down by about 38% in 2010 based on their monitoring data from 13 provinces. Their report also revealed that the drug prices in PHCs were lower than in retail drug stores [19]. Another survey in Haikou, Hainan province showed that drug prices decreased by 28.2% in 2012 compared with the same period before 2009 in six pilot PHCs [20]. Therefore, China’s NEMS has a positive effect on the control of drug prices. Considerable variation in price change was observed in the four provinces studied, which reflected differences in regulatory, economic, and administrative contexts. The major contributing factors reported by previous studies are centralized procurement and zero markup policy, which are two key elements of the NEMS. Centralized procurement helps to reduce the purchasing prices because suppliers are competing for government contracts, and the zero markup policy cancels the profit margins on essential medicines in PHCs, which represents a reduction in retail prices [21–23]. There are also a number of successful examples at the global level. One is the Eastern Caribbean Drug Service, which was established to manage bulk purchasing and competitive tendering on behalf of the member countries of the Organization of Eastern Caribbean States. This service achieved an average of 44% reduction in price during their first tender cycle [24].

One of the most important objectives of NEMS is to bring down drug prices. However, this does not mean that the lower prices are better. An excessive pursuit of lower prices might increase the following risks: a) drugs disappearing from the market because the price was too low, which discourages manufacturers and distributors from supplying the drugs; and b) potential quality risks because the manufacturers may adulterate the drug quality to ensure their profit margin is healthy. A report from three Chinese provinces (Ningxia, Chongqing, and Tianjin) showed that many county officials complained that some manufacturers became reluctant to produce certain drugs because the price controls and recommended procurement procedures in the NEMS severely curtailed their profit margin [23].

### Expensive drugs tended to be more frequently used in the postreform period

The price index expressed price changes in a different way by indicating a price decrease in essential medicines following the implementation of NEMS. Moreover, the difference between *P*^*Pa*^ and *P*^*La*^ showed that the frequently used drugs always had higher prices and milder price reductions in the postreform period. This observation is consistent with the results based on the analysis of individual price differences analysis (Table 4). Similar findings were also reported in another survey in Shandong [25]. The assumption of the NEMS policy design is that drug consumers would prefer drugs to be cheaper. However, this does not consider that drug prescriptions are written by doctors, not by the paying patients. The underlying motivation of doctors to use more-expensive drugs may include: 1) the allocation of government subsidies in many regions (i.e. Anhui, Ningxia) was based on prescription volumes and represented only the lost profit markup. In this case, the irrational incentives for generating revenues from drugs were the same as prereform. 2) The financial incentives given by the manufacturers to healthcare facilities and doctors might have influenced their prescribing behaviors [26]. 3) The profit margin for expensive drugs was relatively higher, which could encourage distributors to guarantee their supply. Because of the lack of stock or the unresponsive supply of lower-priced drugs, doctors had to prescribe the more-expensive drugs.

### PHCs’ financing dilemma

Most PHCs received less revenue from drug prescriptions in the postreform period. In China, drug revenue was the principle source of income for PHCs because the medical revenue cannot support the full operational costs of a PHC. Some PHCs encountered financing dilemmas after the implementation of NEMS. A survey in a city in Zhejiang province performed by the Chinese Academy of Social Sciences showed that the total drug revenue of all PHCs was 186 million yuan in 2009, which included 94 million yuan in profits. The total medical revenue was 70 million yuan, with a profit of 48 million yuan. The government funding was 20.4 million yuan. Therefore, the available net revenue was 162 million yuan in 2009. The salary of health personnel, which is the largest expenditure of PHCs, was 114 million yuan a year. After NEMS, these PHCs experienced financial deficits [27]. A survey in Zhenjiang in Jiangsu province found that 11 of all 13 PHCs experienced a financial deficit in 2011, despite an increase in government funding compared with the prereform period [28]. Furthermore, an investigation in 12 PHCs of Shanghai, Sichuan, and Chongqing indicated the average loss rate of the surveyed PHCs was 5.7% in 2011, which showed an increase of more than four times compared with 1.1% in 2009 [29]. Therefore, there is an acute need to establish a scientific, rational, institutionalized, and standardized compensation system for PHCs. Insufficient compensation may result in shortages in routine operational activities, a weakened ability to maintain their essential medicine system, or sales of diagnostics, technologies, or other revenue-generation methods to cover their basic operational costs [30].

## Limitations

The results of this study should be interpreted in light of several limitations. The present study was conducted shortly after China introduced NEMS. NEMS’s effectiveness and impacts have not yet been fully realized. Furthermore, reforms were still evolving during this study, which means that the timeliness of this study also needs to be updated. In addition, the results of this study were based only on four selected provinces. Although these four provinces were selected to explore different socioeconomic development levels, the conclusions of this study should be cautiously generalized to China. Therefore, there is still room for improvement in updating data, choice of scale, variables, and research method. Countries or other locations can use any indicators in the present study as a baseline assessment for follow-up studies.

## Conclusions

The implementation of NEMS was found to have a positive impact on drug prices in China. However, more-expensive drugs were preferred because of irrational incentives in the postreform period. Furthermore, most PHCs received less revenue from drugs and may experience financing dilemmas after the implementation of NEMS. Policy options such as improvement of the compensation mechanism and rational use of drugs should be promoted further.

## Abbreviations

NEMS: National Essential Medicines System
PHCs: Primary healthcare centers
TCMs: Traditional Chinese medicines
NEDs: National essential medicines
PEDs: Provincial supplemented essential medicines
*P*^*La*^: Laspeyres index
*P*^*Pa*^: Paasche index
US$: United States dollars.
